# Comparison of Healthcare Utilization and Costs Between RA Patients Receiving Biological and Conventional Synthetic DMARDs: A Nationwide Population-Based Cohort Study in Taiwan

**DOI:** 10.3389/fphar.2019.01214

**Published:** 2019-10-22

**Authors:** Der-Yuan Chen, Fun Yu, Li-Wen Tuan, Chao-Hsiun Tang

**Affiliations:** ^1^Rheumatology and Immunology Center, China Medical University Hospital, Taichung, Taiwan; ^2^College of Medicine, China Medical University, Taichung, Taiwan; ^3^Translation Medicine Laboratory, Rheumatology and Immunology Center, China Medical University Hospital, Taichung, Taiwan; ^4^Pfizer Ltd., New Taipei City, Taiwan; ^5^Formosa Biomedical Technology Corp., Taipei, Taiwan; ^6^School of Health Care Administration, Taipei Medical University, Taipei, Taiwan

**Keywords:** rheumatoid arthritis, biologics, disease-modifying antirheumatic drugs, healthcare utilization and costs, Taiwan

## Abstract

**Background:** The therapy with biological disease-modifying anti-rheumatic drugs (bDMARDs) has proven to rapidly reduce articular symptoms/signs, decrease morbidities, and improve health outcome in patients with rheumatoid arthritis (RA) and be cost-effective in Western countries. However, the difference in healthcare utilization and costs between conventional synthetic DMARDs (csDMARDs) and bDMARDs in the treatment of RA patients in Taiwan remains largely unexplored.

**Methods:** Two cohorts of RA patients and their matched controls were identified from the National Health Insurance Research database (NHIRD). The csDMARD cohort comprised of patients who submitted claims during 1997–2003 for cyclosporine≥50 mg/day with concomitant use of ≥2 csDMARDs for ≥28 days (n=1,569), whilst the bDMARD cohort comprised of patients who had ≥1 claim during 2003–2011 for bDMARD (n = 1,530). The per-patient per-year healthcare utilization and costs were estimated by bootstrapping method, with a comparison being undertaken between csDMARD and bDMARD.

**Results:** The incremental number of hospitalization days was reduced from 2.3 days for csDMARD to 0.58 day for bDMARD. When compared to csDMARD-treated patients, the incremental total costs and RA-related medication costs were significantly higher in bDMARD-treated patients (US$9,081 vs. US$2,481; US$8,992 vs. US$1,883). However, the combined incremental healthcare utilization costs and non-RA medication costs were significantly lower in bDMARDs-treated patients compared to csDMARD-treated patients (US$374.7 vs. US$1,156.2).

**Conclusion:** Although total costs increased as a result of introducing biologics in RA treatment, biologics have undoubtedly given rise to the benefits of reduced healthcare utilization. The increase in medication costs from biologics was offset by the lower costs of healthcare utilization. Our findings suggest that the medication costs of biologics may be alleviated by an improvement in clinical outcomes.

## Introduction

Rheumatoid arthritis (RA), a chronic autoimmune disease, has an annual incidence rate of about 0.4% in Taiwan, with females being affected more than males (female:male = 2:1 to 4:1) (Kuo et al., 2013). Dysregulation of immune system in RA results in chronic inflammation of the joints and extra-articular organs. Therefore, RA can lead to persistent inflammation of the affected joints, resulting in joint destruction/disability, a higher risk of cardiovascular disease (CVD), and increased mortality (Avina-Zubieta et al., 2012; Choy et al., 2014). Conventional synthetic disease-modifying antirheumatic drugs (csDMARDs), such as methotrexate (MTX), can relieve the symptoms and delay the progression of RA. Therefore, csDMARDs are recommended as the first-line therapy for RA, either in succession or in a combination with other anti-inflammatory agents (Smolen et al., 2017). However, when there is a decline in treatment efficacy under these regimens, patients usually need alternative therapy; otherwise the disease can become more active and progressive.

Licensed biological agents, comprising of tumor necrosis factor (TNF)-α inhibitors, either monoclonal antibody or immunoglobulin fusion protein, which are grouped as biological DMARDs (bDMARDs), have proven to greatly enhance the effectiveness of RA treatment and improve the health outcomes, in terms of both preventing CVD (Barnabe et al., 2011; Solomon et al., 2013) and reducing mortality (Listing et al., 2015), when compared to those receiving csDMARDs (Smolen et al., 2007; Klareskog et al., 2009). These bDMARDs are available in Taiwan for the treatment of RA patients on whom received at least two csDMARDs (MTX and any one of hydroxychloroquine, sulfasalazine, d-penicillamine, azathioprine, leflunomide, and cyclosporine) according to the guidelines of the British Society for Rheumatology. (Ledingham and Deighton, 2005).

Although the health benefits achieved by the TNF inhibitors are notable, the high price of these agents precludes their widespread prescription and places a financial impact on the healthcare system in Taiwan; thus, csDMARDs, non-steroidal anti-inflammatory drugs (NSAIDs), and corticosteroids continue to play primary roles in the treatment of RA in clinical practice, despite significant numbers of patients showed unsatisfactory responses or intolerance to these therapeutic agents and experienced recurrence of disease activity (Genovese et al., 2002; Voll and Kalden, 2005; Breedveld et al., 2006; Kievit et al., 2011). Among them, poor adherence/persistence or discontinuations are important contributors to treatment failure and disease progression; this, in turn, increases both healthcare utilization and expenditure (Grijalva et al., 2007).

Considering the high price of biologics, numerous studies have reported its cost-effectiveness for RA (Schoels et al., 2010); for example, whilst drug costs have increased among US-employed RA patients since bDMARDs were taken into use, overall medical costs have been reduced (Birnbaum et al., 2012). There is evidence also showing that biologics are associated with cost savings by offsetting the changes in employee utilization of drug and medical services through a reduction of the emergency visits and hospital days, and through an improvement of life quality (Birnbaum et al., 2012).

Similarly, in Taiwan, the annual expenditure on biologics in RA treatment has increased over time (NT$1.11 billion in 2009, NT$1.35 billion in 2010, and NT$1.65 billion in 2011) (National Health Insurance Administration, 2012). However, the overall cost-effectiveness in Taiwan have yet to be fully evaluated; also, there are limited studies estimating the resource utilization of RA patients using real-world data. Along with the first reimbursed bDMARD-etanercept in Taiwan in 2003, the study utilized National Health Insurance Research Database (NHIRD) with longitudinal claim data for the purpose of assessing the impact and cost-effectiveness of bDMARD in Taiwan by comparing the costs and healthcare utilization with csDMARD in the RA treatment.

## Methods

### Study Design

This was a retrospective, epidemiological study aiming to assess the differences in healthcare utilization and costs between in RA patients treated with csDMARDs and bDMARDs, using NHIRD from January 1, 1996, to December 31, 2013. Because biologic was not available in the National Health Insurance (NHI) program until 2003, patients with pharmacy claims of csDMARD or bDMARD were identified from 1996 to 2003, and from 2003 to 2011, respectively. Since csDMARD and bDMARD chohorts were identified from different time period, the comparison between csDMARD and bDMARD was made indirectly to avoid cohort effects. Healthcare utilization and costs were evaluated for the specific categories of outpatient/emergency room visits, RA-related surgery, medication, and ward use.

### Ethic Statements

The independent Ethics Committee/Institutional Review Board at Taipei Medical University approved this study (201209015). The study was conducted in accordance with the applicable local regulations and with the ethical principles laid down in the Declaration of Helsinki. As all personal information was anonymized before analysis, patient consent was not deemed necessary by the Ethics Committee.

### Data Source

The NHIRD is a comprehensive, population-based claims database compiled and maintained by the Taiwan National Health Research Institute. The Taiwan NHI program, launched in March 1995, is a mandatory social health insurance system which covers 99% of more than 23 million people. The dataset consisted of scrambled patient identiﬁcation numbers, gender, date of birth, primary and secondary diagnostic codes, and date, type (outpatient, inpatient, emergency visits), and fees charged of the services provided. The Longitudinal Health Insurance Database (LHID, 2010), which contain all the aforementioned claims records of a 1 million sample cohort representative of the Taiwanese beneficiaries in 2010, were used to identify a matched cohort of csDMARD and bDMARD, respectively.

### Patient Selection

Patients with moderate to severe RA were enrolled during the retrospective study period (1996 to 2011).The diagnosis of RA (International Classification of Diseases, Ninth Revision, Clinical Modification, ICD-9-CM codes 714.0) was made according to the 1987 American College of Rheumatology criteria (Arnett et al., 1988) and the Registry of Catastrophic Illness Patient Database (RCIPD) contained in the NHIRD. The including criteria for the two RA cohorts was as follows. First, for the csDMARD cohort, patients were included if they had medication claim for cyclosporine ≥ 50 mg/day with concomitant use of ≥2 csDMARDs for ≥ 28 days within 56 days from 1997 to 2003, as cyclosporine is recommended for the use in severe RA who have not responded adequately to methotrexate.(Cush et al., 1999). Concomitant csDMARDs considered for this analysis included methotrexate, sulfasalazine, hydroxychloroquine, d-penicillamine, azathioprine, and leflunomide. Second, for the bDMARD cohort, patients were selected if they had ≥ 1 claim for bDMARD from 2003 to 2011. The bDMARDs included etanercept, adalimumab, and rituximab (golimumab, tocilizumab, and abatacept were reimbursed after 2012). The date of the first claim when the patient met the inclusion criteria was defined as the index date.

**Figure 1:**
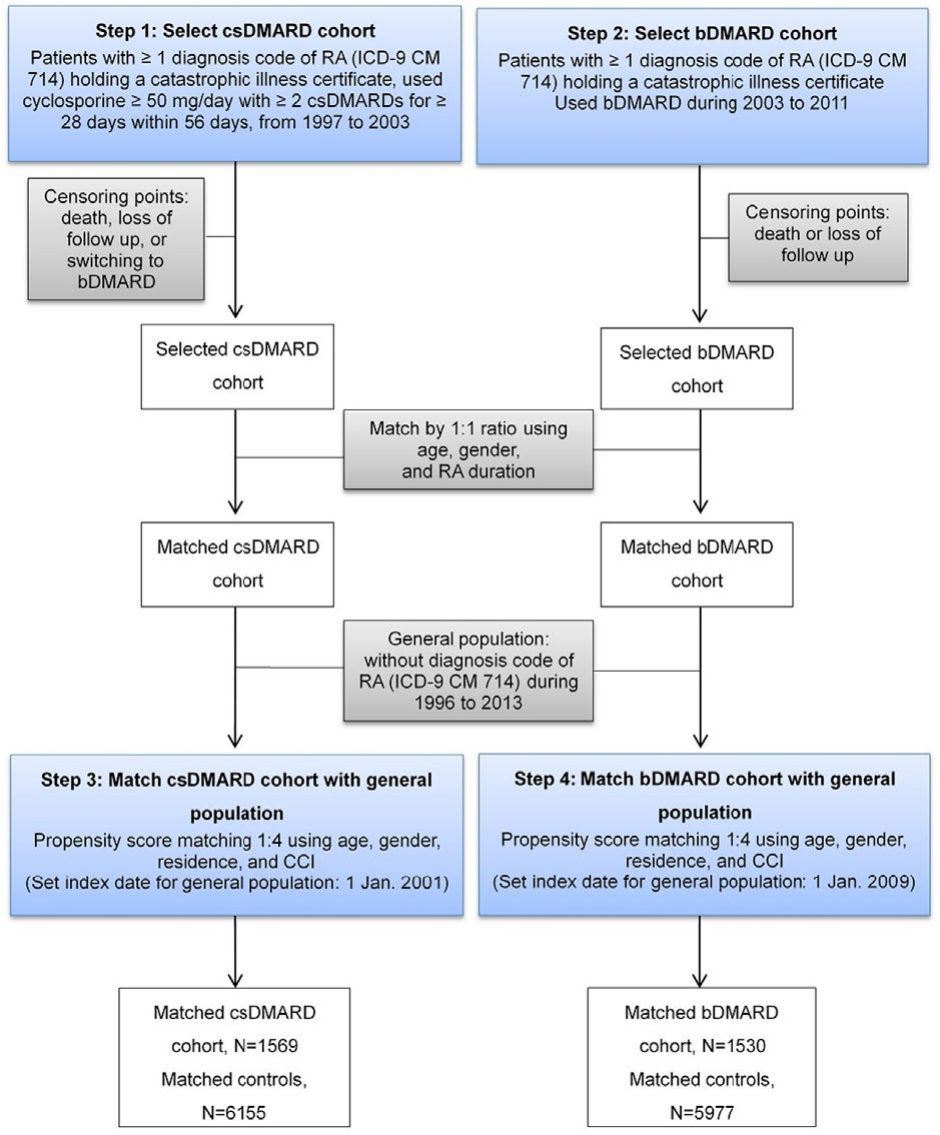
Flowchart of sample selection and population matching.

A two-step approach was applied to ensure patients in csDMARD and bDMARD cohorts were mutually exclusive (**Figure 1**). First, patients who had received csDMARDs during 1997–2003 were selected. These patients were followed-up for at least two years until death, lost follow-up, or switching to bDMARDs, whichever came first. Second, patients who had received bDMARDs during 2003 to 2011 were selected and followed-up for at least two years until death or lost follow-up.

### Population Matching

Because analytic cohorts were not formed by randomization, comparisons between the cohorts could be confounded by a selection bias. To adjust the potential cohort imbalances, csDMARD and bDMARD were matched 1:1 in the first place using age, gender, and RA severity according to RA duration, which was defined as the duration between the index date and the year when the patient had ≥3 claims with RA diagnoses were firstly occurred.

After comparable csDMARD and bDMARD cohorts were determined, propensity score matching (Rosenbaum and Rubin, 1984; D’Agostino, 1998) was performed on the csDMARD and bDMARD cohorts and their respective controls at a ratio of 1:4. The score measures the similarity between RA cases and their controls in terms of a vector of observable characteristics, namely, age, gender, region, and comorbidity profile. Region was defined by the branches of the NHI Administration in which the subjects were enrolled. The comorbidity profile was evaluated using the Charlson Comorbidity Index (CCI), (Romano et al., 1993), a weighted summary measure of important concomitant diseases within one year before the index date, with RA being excluded.

A two-step approach to find the respective matched controls for csDMARD and bDMARD was employed (**Figure 1**). After excluding subjects who had previously been diagnosed with RA (ICD-9-CM code: 714.xx) during 1996 to 2013 in the LHID, 2010 sample cohort files, the matched controls of csDMARD was established first, followed by that of bDMARD, to ensure two control cohorts were mutually exclusive. The index date of the csDMARD and bDMARD was assigned to their respective matched controls.

### Study Measures and Outcomes

#### Baseline Characteristics

Patient characteristics were measured on the index date, with the data including demographic characteristics (age, gender, region, and index year) and RA duration.

#### Healthcare Utilization

All-cause annual healthcare utilization was calculated, including outpatient (OPD) visits, emergency room (ER) visits, number of hospitalizations, and number of hospitalization days.

#### Healthcare Costs

All-cause annual healthcare costs were summarized for the total costs, pharmacy costs, and sub-total costs under various healthcare settings (including OPD, ER, hospitalization, and RA-related surgery). Pharmacy costs were then further divided into RA and non-RA related costs, where RA-related drug costs were those costs associated with csDMARDs or bDMARDs.

### Statistical Analyses

Given that cost data are positive values which follow a non-normal distribution and can also often have zero values, the normality assumption is likely to be invalid due to the skewness of the cost data. We therefore used the non-parametric bootstrapping procedure (Jiang and Zhou, 2004) to carry out the statistical inferences and determine the 95% confidence interval (CI) for per-patient per-year (PPPY) healthcare utilization and costs, with 1,000 non-parametric replications being drawn from the source cohorts. This method estimates the empirical distributional function of the data without imposing any probability density function.

We calculated the PPPY utilization/costs as the sum of the utilization costs for each patient divided by the sum of the total number of days in the observation period for each patient, multiplied by 365 days. All of the costs were adjusted to 2013 US dollars. SAS version 9.4 (SAS Institute Inc., Cary, NC) was used for all of the statistical analyses carried out in this study, with a two-sided alpha level of 0.05 being used to determine the statistical signiﬁcance in all of the comparisons.

## Results

### Patient Characteristics

After applying the study eligibility criteria and population matching, as shown in **Table 1**, we identified a total of 1,569 patients in the csDMARD cohort versus 6,155 in the csDMARD control cohort, and 1,530 patients in the bDMARD cohort versus 5,977 in the bDMARD control cohort. No significant differences in age, gender, and region were found between csDMARD cohort and csDMARD control, or between bDMARD cohort and bDMARD control.

**Table 1:** Clinical and demographic characteristics.

Variables	csDMARD Comparison				**bDMARD Comparison**	
	csDMARD N = 1569	csControl N = 6155		bDMARD N = 1530		bControl N = 5977
Age (years), mean ± SD	51.5 ± 12.8	51.8 ± 13.1		51.4 ± 12.9		51.5 ± 13.4
Gender, No. (%)						
Male	354 (22.6)	1385 (22.5)		342 (22.4)		1350 (22.6)
Female	1215 (77.4)	4770 (77.5)		1188 (77.6)		4627 (77.4)
Region, No. (%)						
Northern	451 (28.7)	1826 (29.7)		741 (48.4)		2867 (48.0)
Central	667 (42.5)	2531 (41.1)		379 (24.8)		1466 (24.5)
Southern	419 (26.7)	1670 (27.1)		377 (24.6)		1511 (25.3)
Eastern	32 (2.0)	128 (2.1)		33 (2.2)		133 (2.2)
RA duration (years), mean ± SD	1.9 ± 1.5	–		2.1 ± 1.4		–
Index period (years), mean ± SD	7.4 ± 3.1	9.9 ± 0.5		4.9 ± 2.3		5.0 ± 2.3

RA, rheumatoid arthritis; csDMARD, conventional synthetic disease-modifying antirheumatic drug; bDMARD, biologic disease-modifying antirheumatic drug; SD, standard deviation; CCI, Charlson Comorbidity Index.

### All-Cause Healthcare Utilization

A summary of the annual all-cause healthcare utilization following the application of bootstrapping is provided in **Table 2**. RA patients were found to have significantly higher numbers of OPD visits and hospitalizations than the general population, although the incremental numbers were comparable between cSDMARD and bDMARD treatment, as compared to the general population.

**Table 2:** All-cause annual healthcare utilization per person per year comparison among DMARD and non-RA control using bootstrapping.

Category	csDMARD Comparison, mean (patient-year)			bDMARD Comparison, mean (patient-year)		
	csDMARD (95% CI*)	csControl (95% CI*)	Difference	bDMARD (95% CI*)	bControl (95% CI*)	Difference
Outpatient visits	31.9 (30.9-32.8)	22.3 (22.0-22.7)	9.50	32.5 (31.5-33.4)	23.1 (22.7-23.6)	9.30
Emergency room visits	0.22 (0.2-0.2)	0.16 (0.2-0.2)	0.06	0.31 (0.3-0.3)	0.29 (0.3-0.3)	0.02
Admissions	0.44 (0.4-0.5)	0.18 (0.2-0.2)	0.26	0.48 (0.4-0.5)	0.25 (0.2-0.3)	0.23
Hospitalization days	3.9 (3.5-4.3)	1.6 (1.3-1.8)	2.30	3.2 (2.8-3.5)	2.6 (2.1-3.0)	0.58
RA-related surgery	0.039 (0.03-0.04)	0.007 (0.006-0.007)	0.03	0.031 (0.03-0.04)	0.007 (0.006-0.008)	0.02

*indicates empirical-bootstrapping confidence interval.

As shown in **Table 2**, the number of incremental hospitalization days was reduced from 2.3 days for csDMARD to 0.58 day for bDMARD. The length of hospitalization stay was comparable between RA patients using bDMARD and the general population, but significantly longer for RA patients on csDMARD than that for the general population (csControl).

### All-Cause Healthcare Costs

As illustrated in **Table 3**, total PPPY costs of the healthcare resources greatly increased for RA patients, compared to the general population, particularly for the bDMARD cohort (total incremental costs: bDMARD vs. csDMARD = US$9,081 vs. US$2,481). The major difference was found in the medication costs, which accounted for 75.9% (US$1,883) of the total incremental costs for csDMARD, and 99.0% (US$8,992) of the total incremental costs for bDMARD. As regards the total RA patient costs, bDMARD costs accounted for a high share up to 79.4% (US$8,712), whereas csDMARD costs accounted for 35.3% (US$1,327). Moreover, the incremental hospitalization costs fell by 53.5%, from US$457.5 for csDMARD to US$212.6 for bDMARD. Increments in RA-related surgery costs fell by 34%, from US$139.2 for csDMARD to US$91.9 for bDMARD (**Table 3**).

**Table 3:** All-cause healthcare costs per patient per-year comparison among DMARD and non-RA control using bootstrapping.

Category	csDMARD Comparison, mean ± SD (US$/patient-year)			bDMARD Comparison, mean ± SD (US$/patient-year)		
	csDMARD (95% CI*)	csControl (95% CI*)	Difference	bDMARD (95% CI*)	bControl (95% CI*)	Difference
Total costs	3,757 ± 69.0	1,276 ± 31.1	2,481	10,975 ± 119.2	1,894 ± 50.8	9,081
	(3,623-3,897)	(1,216-1,336)		(10,746-11,200)	(1,793-1,993)	
Outpatient visits	784.7 ± 25.2	644.5 ± 23.6	140.2	830.0 ± 25.1	953.9 ± 35.0	-124
	(733.2-828.1)	(598.1-691.3)		(777.7-877.2)	(883.3-1,021)	
Admissions	723.1 ± 34.2	265.6 ± 10.7	457.5	632.9 ± 36.2	420.3 ± 17.4	212.6
	(659.3-793.2)	(242.7-285.2)		(562.2-701.0)	(385.6-454.0)	
Emergency room visits	22.8 ± 1.5	16.5 ± 0.6	6.3	33.8 ± 2.0	33.9 ± 1.3	-0.07
	(19.6-25.5)	(15.3-17.5)		(29.6-37.5)	(31.1-36.4)	
RA-related surgery	162.2 ± 10.2	23.0 ± 1.5	139.2	115.5 ± 11.1	23.5 ± 2.3	91.9
	(142.9-182.4)	(19.8-25.6)		(92.5-135.2)	18.7-27.6)	
Total medication costs	2,249 ± 39.6	366.7 ± 8.7	1,883	9,514 ± 107.8	521.6 ± 17.7	8,992
	(2,172-2,322)	(349.5-384.2)		(9,305-9,725)	(485.7-554.9)	
RA medication	1,327 ± 30.5	3.2 ± 1.0	1,324	8,712 ± 107.8	5.6 ± 1.6	8,707
	(1,265-1,380)	(0.9-4.8)		(8,510-8,921)	(2.2-8.2)	

*indicates empirical-bootstrapping confidence interval.

**Figure 2:**
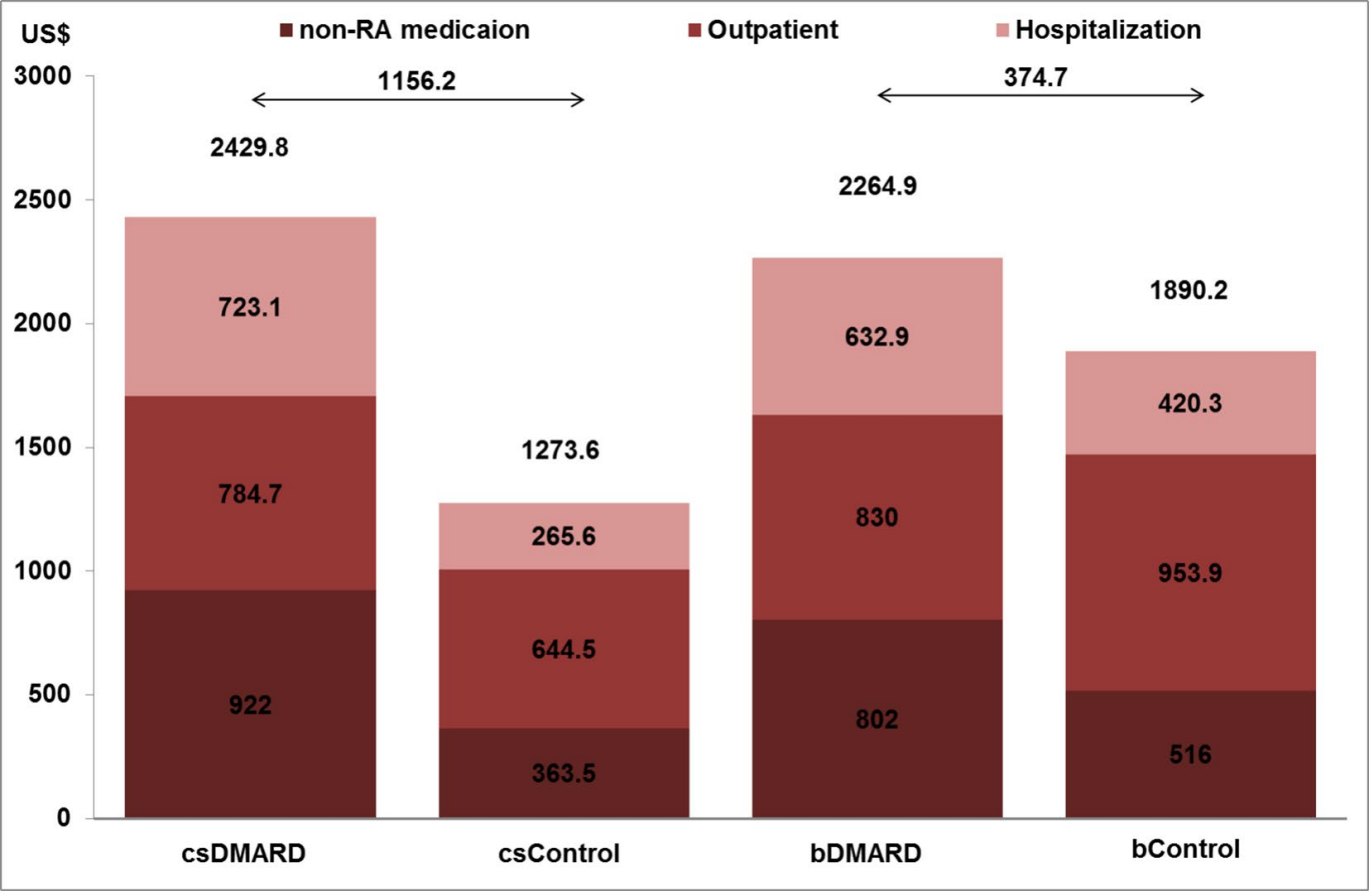
Healthcare system and non-RA medication costs and total medication costs per patient year for RA and non-RA patients.

As shown in **Figure 2**, the outpatient costs were increased with csDMARD but reduced with bDMARD (188% reduction, from US$140.2 to –US$124). The incremental non-RA medication costs were reduced by 48.8%, from US$558.5 for csDMARD to US$286 for bDMARD. Finally, despite the increased medication used costs in bDMARD, total healthcare utilization in combination with non-RA medication costs were reduced by 67.6%, from US$1,156.2 for csDMARD to US$374.7 for bDMARD.

## Discussion

This nationwide study, using a cross-sectional database from the NHI program, firstly described the clinical outcomes and patterns of direct medical costs in RA patients using DMARDs (traditional or biological) as compared to the general population with non-RA over a long-term observation period, running from 1996 to 2013 in Taiwan. We demonstrated that the use of bDMARD reduced healthcare resources by shortening the length of hospitalization stay (1.72 days shorter) and a reduction of the healthcare utilization costs when compared to csDMARD, and resulted in the reduction of the incremental costs by 67.6% (csDMARD vs. bDMARD: US$1,156.2 vs. US$374.7).

Various types of biologics have been introduced over the past decade to effectively treat RA patients; however, whilst this treatment regimen has clearly reduced health care utilization, it continues to represent a financial impact on the NHI program. Our analysis shows that bDMARDs have substantially increased the total costs of RA patients through a three-fold increase in total costs; bDMARD medication costs accounted for almost 80% of the total costs, as compared to csDMARDs, which accounted for only 35.3% of the total costs. The ﬁnancial impacts of bDMARD adoption were also reported in other countries; for example, a French observational study reported that annual medical costs had increased almost three-fold after the introduction of etanercept (Juillard-Condat et al., 2008), whilst another study reported a three-fold increase over the prior year in healthcare costs after the introduction of biologics, due to the increased drug costs (Johansson et al., 2015).

Despite the increased medication costs, some studies reveal that bDMARDs have been proven to have cost-saving effects on healthcare. Consistent with our analyses, a retrospective analysis of a large US claims database found that all-cause healthcare costs were higher in patients receiving csDMARDs prior to the introduction of bDMARDs (Betts et al., 2016). The difference was US$772 for patients using 1 vs. 2 csDMARDs and US$2,390 for patients using 2 vs. 3 csDMARDs. An overview of the differences in the healthcare utilization and costs for RA patients between 1997 and 2006 also concluded that biologics may be associated with cost savings by offsetting the changes in drug expenditure; the specific cost savings identified were reductions in medical services, including hospital days and emergency visits (Birnbaum et al., 2012). When compared to 1997, annual drug costs had increased by US$633 per patient by 2006, but medical costs had fallen by US$618 per patient. The cost-saving effects of bDMARD were also reflected in clinical outcomes such as reduced incidences of CVD, comorbidity, and mortality rates (Pappas et al.,; Barnabe et al., 2011; Solomon et al., 2013). Along with the reduced healthcare utilization in our analysis, the clinical benefits suggest a possible association with an improved control of RA and its comorbidities under bDMARD therapy.

Conversely, csDMARDs-treated patients were found to incur higher healthcare costs. These patients might have suboptimal responses, which may cause additional clinical and economic burdens (Kotak et al., 2013). These burdens were not only limited to direct medical costs but also the indirect costs incurred by society as a result of lost productivity and reduced patient and family incomes, since it has been reported that patients with moderate disease activity were more likely to be unemployed due to disability (Kotak et al., 2013).

Furthermore, RA frequently leads to presenteeism, the cost of which is usually higher than medical costs (Olsson et al., 2004), whilst the use of multiple csDMARDs has been reported to be associated with joint damage due to inadequate therapeutic response and some signiﬁcant side effects (O’Dell et al., 2013). This implies that the increased healthcare utilization incurred by csDMARDs, which in turn, may be associated with a higher disease burden, resulting in reduced productivity at work and an increase in indirect social costs. On the other hand, the improved clinical outcomes such as articular symptoms/signs, the duration of morning stiffness, and fatigability under bDMARDs treatment may well offset these indirect social costs.

The strength of our study is in the utilization of the NHIRD from the Taiwan reimbursement system, which provides universal healthcare coverage for 99% of the Taiwan population. Based on its distinguishing comprehensive data and long observational period, the NHIRD is an ideal data source for epidemiologic research. Furthermore, our research represents not only a cross-sectional study, but also a longitudinal study, since it spans lengthy study periods of up to 10 years. The longitudinal data provide an opportunity to detect changes or developments in the characteristics of the target population as well as the long-term influence of RA management during the DMARD transition period, from traditional treatment to new biologics, thereby identifying sequences of events.

However, a few limitations of this study should be addressed. Firstly, with data from a different era, it was difficult to directly compare healthcare utilization under traditional treatment with that under biologics due to differences in patient populations and reimbursement policies, improvements in medical care, and currency inflation. We therefore used an indirect means of comparing RA patients over these different periods by comparing the samples with their matched non-RA population over the same sample period. Even so, given that the impact of the above factors may, to some extent, have influenced our results, it cannot be ignored. Secondly, by using claims data, we were unable to evaluate the indirect social costs attributable to different RA management. To achieve this, we would need to be able to investigate productivity losses from employer perspectives to see whether RA and its comorbidities can lead to a reduction in work productivity, and whether substantial medication expenses after the introduction of bDMARD may have eased such reduced productivity. Finally, the comorbidities were identiﬁed using ICD-9-CMcodes that are used for administration purposes; however, certain comorbidities may be underestimated. Furthermore, the surgery codes were not validated, so it is possible that some patients underwent RA-related surgery for other diseases.

In conclusion, although total costs have been increased with the introduction of biologics in RA treatment, bDMARDs potentially resulted in the benefit of reduced healthcare utilization; as such, the increase in medication costs from biologics seems to have been offset by the reduced costs of healthcare utilization, which suggests that the medication costs of biologics may well be offset by an improvement of clinical outcomes.

## Data Availability Statement

All datasets generated for this study are included in the article/supplementary material.

## Ethics Statement

As a requirement of publication, the authors have provided the publisher with a signed confirmation of compliance with legal and ethical obligations, including, but not limited to, the following: authorship and contribution, conflicts of interest, privacy and confidentiality, and where applicable, protection of human and animal research subjects. The authors have read and confirmed their agreement with the ICMJE authorship and conflict of interest criteria. The authors also confirm that this article is unique and not under consideration or published in any other publication and that they have permission from rights holders to reproduce any copyrighted material. Any disclosures are made in this section.

## Author Contributions

The contributions made by the individual authors are as follows. D-YC: study design and results interpretation. C-HT: study design, data collection, and data analysis. FY: data analysis. L-WT: data analysis. All authors have participated in manuscript writing and editing.

## Funding

This study was funded by Pfizer Ltd., Taiwan. The Funder had the following roles in the following: data analysis, decision to publish, preparation of the manuscript. FY is the employee of Pfizer Taiwan.

## Conflict of Interest

This study was funded by Pfizer Ltd., Taiwan. The Funder had the following roles in the following: data analysis, decision to publish, preparation of the manuscript. FY is the employee of Pfizer Taiwan. The remaining authors declare that the research was conducted in the absence of any commercial or financial relationships that could be construed as a potential conflict of interest.
